# Optimizing Piezoelectric Nanocomposites by High‐Throughput Phase‐Field Simulation and Machine Learning

**DOI:** 10.1002/advs.202105550

**Published:** 2022-03-11

**Authors:** Weixiong Li, Tiannan Yang, Changshu Liu, Yuhui Huang, Chunxu Chen, Hong Pan, Guangzhong Xie, Huiling Tai, Yadong Jiang, Yongjun Wu, Zhao Kang, Long‐Qing Chen, Yuanjie Su, Zijian Hong

**Affiliations:** ^1^ School of Optoelectronic Science and Engineering University of Electronic Science and Technology of China Chengdu 610054 P. R. China; ^2^ School of Materials Science and Engineering The Pennsylvania State University University Park PA 16802 USA; ^3^ School of Computer Science and Engineering University of Electronic Science and Technology of China Chengdu 610054 P. R. China; ^4^ Lab of Dielectric Materials School of Materials Science and Engineering Zhejiang University Hangzhou 310027 P. R. China

**Keywords:** high‐throughput phase‐field simulation, machine learning, nanocomposites, piezoelectric coefficient

## Abstract

Piezoelectric nanocomposites with oxide fillers in a polymer matrix combine the merit of high piezoelectric response of the oxides and flexibility as well as biocompatibility of the polymers. Understanding the role of the choice of materials and the filler‐matrix architecture is critical to achieving desired functionality of a composite towards applications in flexible electronics and energy harvest devices. Herein, a high‐throughput phase‐field simulation is conducted to systematically reveal the influence of morphology and spatial orientation of an oxide filler on the piezoelectric, mechanical, and dielectric properties of the piezoelectric nanocomposites. It is discovered that with a constant filler volume fraction, a composite composed of vertical pillars exhibits superior piezoelectric response and electromechanical coupling coefficient as compared to the other geometric configurations. An analytical regression is established from a linear regression‐based machine learning model, which can be employed to predict the performance of nanocomposites filled with oxides with a given set of piezoelectric coefficient, dielectric permittivity, and stiffness. This work not only sheds light on the fundamental mechanism of piezoelectric nanocomposites, but also offers a promising material design strategy for developing high‐performance polymer/inorganic oxide composite‐based wearable electronics.

## Graphical abstract

A high‐throughput phase‐field model is built to systematically investigate the influence of oxide filler morphology on the piezoelectric properties of polymer/oxide nanocomposites. It is discovered that with a constant filler volume fraction, the vertical pillars exhibit the best piezoelectric performances. A linear regression‐based machine learning model is further employed to predict the performance of the polymer/ceramic nanocomposites.

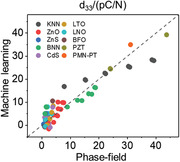

## 1 Introduction

With the gradual emergence of the internet of things (IoT) and smart wearable industry revolution, piezoelectric materials with the intriguing ability for mechanical‐electrical energy conversion have attracted considerable attention in the fields of robotics, ^[^ ^1^, ^2^, ^3^, ^4^, ^5^ ^]^ human‐machine interaction (HMI),^[^ ^6^, ^7^, ^8^, ^9^, ^10^ ^]^ energy harvesters,^[^ ^11^, ^12^, ^13^, ^14^, ^15^, ^16^, ^17^ ^]^ and mobile personalized healthcare,^[^ ^18^, ^19^, ^20^, ^21^, ^22^, ^23^, ^24^ ^]^ etc. Multifunctional capability, flexibility, miniaturization, and self‐powered operation are desired attributes for wearable electronics and implanted devices. In order to achieve high‐performance on‐body electronics, advanced soft piezoelectric materials with stretchability, biocompatibility, and high energy conversion efficiency are demanded.

Inorganic piezoelectric oxides such as BaTiO_3_ (BTO),^[^ ^25^ ^]^ PbZr_x_Ti_1‐x_O_3_ (PZT),^[^ ^26^ ^]^ and (1‐0.35)PbMg_1/3_Nb_2/3_O_3_‐0.35PbTiO_3_ (PMN‐35PT),^[^ ^27^, ^28^ ^]^ etc. possess high piezoelectricity (typically in the order of 100 pC N^−1^) and excellent electromechanical coupling efficiency (can reach 40 %–50 %). Meanwhile, they suffer from several drawbacks such as rigidity, brittleness, and inferior compatibility with the human body^[^ ^29^ ^]^, which limits the applications in soft, wearable electronics. In comparison with the inorganic piezoelectric oxides, the piezoelectric polymers, such as PVDF, PVDF‐TrFE, and P(VDF‐HFP)^[^ ^30^, ^31^, ^32^, ^33^, ^34^, ^35^ ^]^ are mechanically flexible and bio‐compatible but exhibit weak piezoelectricity (typically 10–30 pC N^−1^). Therefore, a combination of high piezoelectricity inorganic piezoelectric oxides and high flexibility organic polymer matrixes offers a feasible route to overcome these shortcomings and synergize the merits of the polymers and piezoelectric oxides. The emergence of ceramic‐polymer nanocomposites has greatly improved the output performance of flexible piezoelectric devices.^[^ ^36^ ^]^ In addition, composite films prepared by processes such as casting and electrospinning have a simpler manufacturing route than ceramic nanowires that require microstructure processing techniques such as photolithography.^[^ ^37^ ^]^ Therefore, nanocomposites are favorable for the large‐scale application of piezoelectric materials in varieties of fields. For instance, by virtue of surface modification and material engineering strategies,^[^ ^38^ ^]^ piezoelectric composites endow multifunctional capabilities in a wide variety of applications including sensing, actuation, energy acquisition, and catalysis. The interfacial coupling between the oxide fillers and polymer matrixes is the main factor in determining the electromechanical coupling efficiency of nanocomposites during the energy conversion procedure.^[^ ^13^, ^35^ ^]^ However, the impact of geometrical morphologies and spatial orientation as well as material constants of the oxide fillers on the effective properties of nanocomposites have not been systematically investigated, which poses a huge challenge for the designing and preparation of high‐performance piezoelectric nanocomposites.

In this work, high‐throughput phase‐field simulations together with machine learning are employed to investigate the effective stress transfer efficiency, effective dielectric permittivity, and piezoelectric coefficient of polymer/ceramic nanocomposites. Previously, the integration of the high‐throughput phase‐field method with machine learning has been widely applied to design high‐energy‐density polymer/ceramic nanocomposites.^[^ ^39^, ^40^, ^41^ ^]^ Herein, it is discovered that with a constant volume fraction, the piezoelectric coefficient and dielectric permittivity increase monotonically with the depth‐to‐width ratio (DR) of fillers, while the mechanical compliance follows an opposite trend. Among 400 geometric configurations, the 1–3 composites with nanopillars perpendicular to the film plane possess the optimal properties of piezoelectric coefficient, electromechanical coupling efficiency (*k*_33_), and quality factor (*d*_33_/*c*_33_, *k*_33_, *d*_33_ *s*_33_). Furthermore, a machine learning strategy is adopted to model and predict the performance of PVA‐polymer (polyvinyl alcohol) nanocomposite composed of various oxide fillers. The present work not only strengthens the fundamental understanding of the influence of fillers on the piezoelectric behaviors but also paves the way for designing and optimizing high‐performance piezoelectric composites.

## 2 Results and Discussion

**Figure 1:**
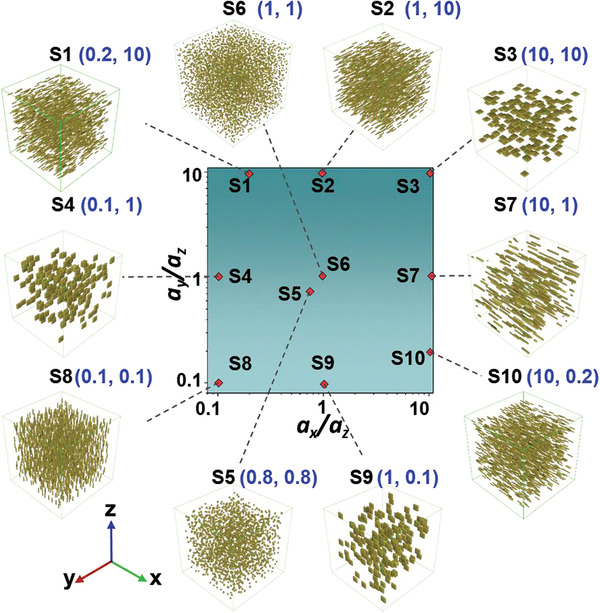
Range of the geometric ratios *a*_y_ */a*_z_ and *a*_x_ */a*_z_ of oxide fillers of the computing dataset and schematics of selected architectures.

We first investigate the effect of the topological shape and orientation of oxide fillers on the stress transfer ability and effective dielectric permittivity as well as the electromechanical coupling efficiency of polymer/ceramic nanocomposites. High‐throughput phase‐field simulations are performed to analyze the effective properties of the BTO/PVA composites with versatile geometrical morphologies to design the optimal configuration with both excellent piezoelectric and mechanical properties. In this study, we choose PVA as the polymer matrix because PVA has good degradability,^[^ ^42^ ^]^ stretchability,^[^ ^43^ ^]^ biologically compatibility,^[^ ^44^ ^]^ and self‐healing ability,^[^ ^45^ ^]^ which makes it an excellent candidate material for biomedical applications. For the high‐throughput calculations, a set of 400 (20 × 20) composite architectures are generated simultaneously to form a computing dataset by tuning the geometric ratios *a*_y_/*a*_z_ and *a*_x_ */a*_z_ of oxide fillers ranging from 0.1–10, assuming that the oxide fillers randomly disperse in the polymer matrix with a constant volume fraction of 1 vol%. Ten representative filler geometrical morphologies are selected from the dataset in order to efficiently reflect the structure discrepancy of the oxide fillers, as shown in **Figure** **1**.

In order to quantitatively understand the piezoelectric and mechanical performance of the nanocomposites with diverse geometric ratios, a comprehensive phase‐field simulation is performed to systematically study the mechanical, electrical, and piezoelectric fields for various architectures. In the phase‐field model, the simulation system is divided into a three‐dimensional array of 128 × 128 × 128 grid points with three‐dimensional periodic boundary conditions for the stress, strain, and electric field.^[^ ^46^, ^47^ ^]^ Since we are only interested in the equilibrium‐state material response, in this work, we build a quasi‐static model where the responses can be obtained by directly solving the mechanical and electrical equilibrium equations. The spatial distribution of electric field **E**(**r**), polarization **P**(**r**), stress ***σ*** (**r**), and strain ***ε*** (**r**) of the composites in response to external stress can be attained by solving the following equations via the Fourier‐spectral iterative‐perturbation method,^[^ ^48^, ^49^ ^]^

(1) $$\nabla \cdot D\;=\;\nabla \cdot \;\left(\varepsilon_{0}\varepsilon_{r}E+d\sigma \right)=\;0$$

(2) $$\nabla \cdot \sigma \;=\;\nabla \cdot \;\left(c\varepsilon -cd^{T}E\right)=\;0$$

where *ε*_0_ is the vacuum permittivity, ***ε***_r_ is the relative dielectric constant of the local phase, **c** is the elastic stiffness, and ***d*** is the piezoelectric coefficient. The volumetric average of the stress tensor $\bar{\sigma }$ is set to the given applied stress ****σ****_app_, i.e.,

(3) $$\;\bar{\sigma }=\;\sigma_{\mathrm{app}}=\left[\begin{array}{ccc} 0 & 0 & 0\\ 0 & 0 & 0\\ 0 & 0 & 1 \end{array}\right]\;\left(\mathrm{MPa}\right)\;$$

It should be noted here that the interfacial effect is neglected for the sake of simplicity, where the full transfer of stress and electric field across the matrix‐filler interfaces is assumed. The materials constants for the BTO filler and PVA matrix are listed in Table S1 (Supporting Information).

**Figure 2:**
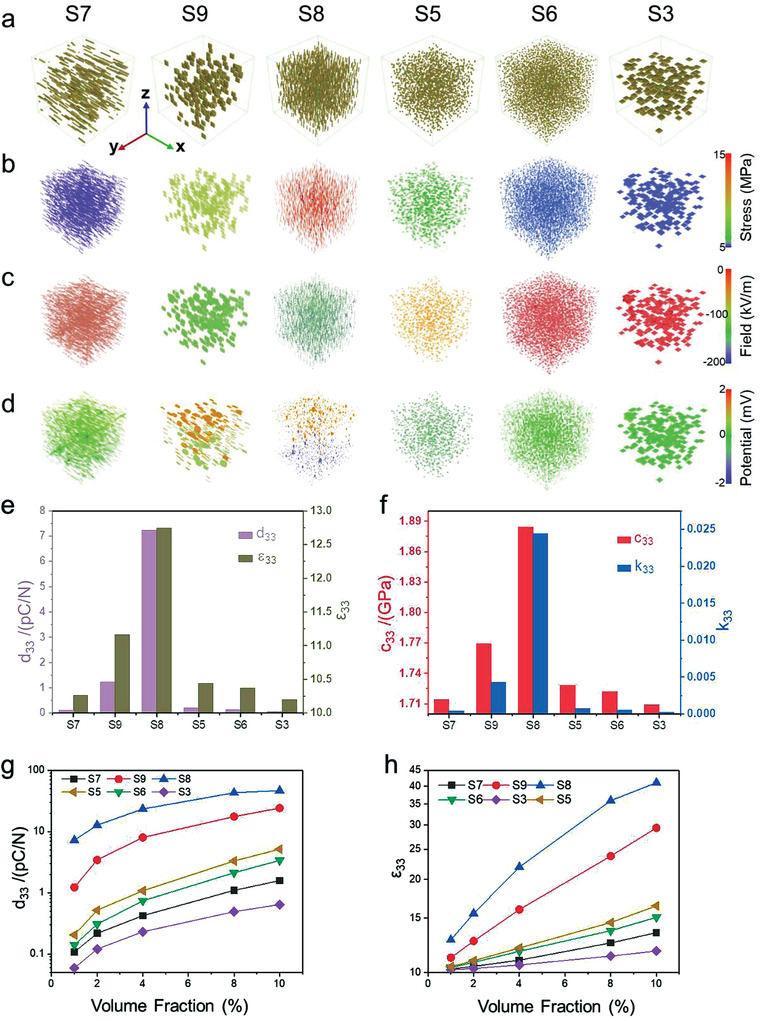
Phase‐field simulation of 6 types of nanocomposites with different filler configurations. a) Structure of the selected types of nanocomposites with 1 vol% filler fraction. b) corresponding stress, c) electric field, and d) electric potential distribution within the nanofillers in response to an applied stress of 1 MPa along the *z*‐axis of the above nanocomposites. e–f) Piezoelectric coefficient *d*_33_, relative permittivity *ε*_33_, elastic stiffness *c*_33_, and the electromechanical coupling coefficient *k*_33_ of the above nanocomposites. g) Effective piezoelectric coefficient and h) dielectric permittivity for the 6 types of nanocomposites with filler fractions ranging 1–10 vol%.

As shown in **Figure** **2**a, 6 types of nanocomposites composed of randomly aligned nanofillers with diverse shapes and orientations are constructed by computer, including horizontal pillar (S7), vertical sheet (S9), vertical pillar (S8), vertical rod (S5), sphere (S6), and horizontal sheet (S3) at a fixed 1 vol%. Their corresponding stress distribution, piezoelectric field, and electric potential in response to applied stress of 1 MPa along the *z*‐axis are visualized in Figure 2b–d, respectively.

Notably, the vertical pillar (S8) and sheet (S9) demonstrate larger and more inhomogeneous stress distribution among all the structures investigated in this study, implying a superior stress transfer efficiency. It is worth noting that both the vertical pillar and the vertical sheet exhibit larger stress inside the oxide fillers than at the oxide‐polymer interfaces. This indicates that the mismatch of the elastic moduli between the flexible polymer and the rigid piezoelectric oxides at the interface imposes a tremendous obstacle for transferring the net mechanical stress, while the large and homogeneous elastic stiffness inside the oxide fillers is conducive to the applied stress delivery. As a result, the applied stress induces a larger electric field and electric potential within the vertical pillar than other structures like sphere, horizontal sheet, and horizontal pillar. Figure S1 (Supporting Information) illustrates the stress distribution, piezoelectric field, and electric potential for the other four geometric configurations (S1, S2, S4, S10). Among the 10 representative structures (Figure 2e,f), the vertical pillar with the smallest *a*_x_ */a*_z_ and *a*_y_ */a*_z_ shows the maximum piezoelectric coefficient (*d*_33_), mechanical stiffness (*c*_33_) and relative permittivity (*ε*_33_), as well as the electromechanical coupling coefficient (*k*_33_).

To evaluate the impact of filler volume fraction on the effective properties of piezoelectric nanocomposites, we perform a systematic study for the piezoelectric coefficient, dielectric permittivity, and elastic stiffness of polymer nanocomposites as a function of filler volume fractions, with results presented in Figure 2g,h. Apparently, both the effective piezoelectric coefficient and dielectric permittivity increase with increasing filler volume fraction from 1 to 10 vol%. For all filler volume fractions, the *d*_33_, *ε*_33_, *k*_33_, and *c*_33_ (see Figure S2, Supporting Information) show a dependence on the type of geometric architectures (S7, S9, S8, S5, S6, S3) with a similar pattern to that of the nanocomposite at 1 vol%, indicating a consistent effect of the geometric modulation. Figure S3 (Supporting Information) visualizes the mechanical and piezoelectric properties for 5 typical geometrical morphologies (S8, S6, S3, S7, and S9) of polymer nanocomposites at a fixed volume fraction of 4 vol%. The nanocomposite with 4 vol% BTO exhibits stronger geometric architecture dependency as compared to the case with smaller BTO content (e.g., 1 vol%), in which the vertical pillar exhibits a higher electric potential and field distribution as compared to the other architectures in this study, indicative of an ideal configuration for energy transduction and self‐power detection.

**Figure 3:**
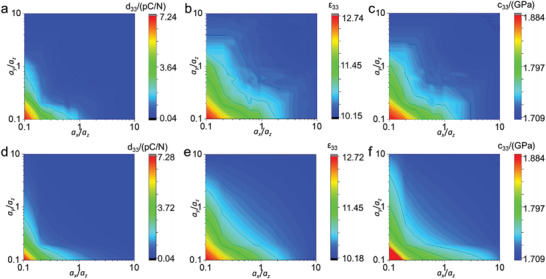
High‐throughput phase‐field simulation results of the whole dataset of the nanocomposites, including a) piezoelectric coefficient *d*_33_, b) relative permittivity *ε*_33_, and c) elastic stiffness *c*_33_ with various filler geometries. Machine learning results of d) piezoelectric coefficient, e) dielectric permittivity, and f) mechanical stiffness.

To comprehensively illustrate the high‐throughput simulation for the whole dataset (20 × 20) of the nanocomposites, we calculate the piezoelectric, mechanical, and dielectric properties of versatile architecture with various geometrical ratios via phase‐field modeling, as shown in **Figure** **3**. Evidently, the effective piezoelectric coefficient (*d*_33_), dielectric permittivity (*ε*_r_), and elastic stiffness (*c*_33_, *c*_44_) varies continuously with the variation of geometrical ratios, i.e., *a*_y_ */a*_z_ and *a*_x_ */a*_z_. With the microstructure of nanofiller changing from horizontal nanosheet (S3, right‐top corner) to vertical pillar (S8, left‐bottom corner) along the diagram diagonal, the piezoelectric coefficient (*d*_33_) and the effective dielectric permittivity (*ε*_r_) of the nanocomposite increases monotonically from 0.059 to 7.240 pC N^−1^ and from 10.20 to 12.74, respectively, showing a strong dependence on the filler geometrical ratios under a constant volume fraction. It is worth noting that the effective stiffness follows a similar tendency to the piezoelectric coefficient, which indicates that high piezoelectricity and high flexibility is incompatible for the nanocomposites in this study.

To further optimize and guide the design and preparation of the piezoelectric oxides/polymer‐based nanocomposites, a facile and predictive model based on regression is proposed to achieve a comprehensive analytical expression for piezoelectric and mechanical properties as a function of geometric ratios of the fillers. Two variables, namely the geometric ratios *a*_x_ */a*_z_ and *a*_y_ */a*_z_ are employed as the main fingerprints for the machine learning model. The machine learning results on the piezoelectric coefficient, dielectric permittivity, and mechanical stiffness with the geometry variation are elucidated in Figure 3d–f, respectively. Notably, all the machine learning results of the material properties agree well with the phase‐field simulation results, showing the accuracy and reliability of the regression‐based machine learning. The relationship of the piezoelectric coefficient (*d*_33_) with respect to the geometric ratios can be expressed as,

(4) $$\begin{array}{ccc} d_{33} & = & \frac{\begin{array}{c} 0.1738-0.00347\ln x-0.00435\ln y-0.00231\ln^{2}x\\ \;-\;0.00232\ln^{2}y+0.00881\ln x\ln y \end{array}}{\begin{array}{c} 1+0.39875\ln x+0.39438\ln y+0.00534\ln^{2}x\\ \;+\;0.00434\ln^{2}y+0.1513\ln x\ln y \end{array}}\\ & & \times \;\left(\mathrm{pC}\;N^{-1}\right) \end{array}$$

where *x* and *y* refer to *a*_x_ */a*_z_ and *a*_y_ */a*_z_, respectively. This expression gives a coefficient of determination of *R* ^2^ = 0.99388707. Note that it is a monotonically decreasing function of *a*_x_ */a*_z_ and *a*_y_ */a*_z_ in the range (0.1, 10) with a maximum value at (0.1, 0.1), indicating that the piezoelectric coefficient of polymer nanocomposite can be improved by enhancing the length‐to‐width ratio of oxide fillers along the longitudinal direction. On the other hand, the expressions of relative dielectric permittivity and mechanical stiffness endow a coefficient of determination value of *R* ^2^ = 0.9809938061 and 0.99030566, respectively, and can be attained as

(5) $$\varepsilon_{33}=\frac{\begin{array}{c} 10.44+3.760\ln x+3.742\ln y+0.06738ln^{2}x\\ \;+\;0.04148ln^{2}y+1.3096\ln x\ln y \end{array}}{\begin{array}{c} 1+0.3706\ln x+0.3689\ln y+0.00846ln^{2}x\\ \;+\;0.00617ln^{2}y+0.1282\ln x\ln y \end{array}}$$

(6) $$c_{33}=\frac{\begin{array}{c} 1.724+0.3275\ln x+0.2059ln^{2}x-0.1756ln^{3}x\\ \;+\;0.53055\ln y+2.366 \end{array}}{1+0.1897\ln x+0.1189ln^{2}x+0.31124\ln y}\;\left(\mathrm{GPa}\right)$$

Furthermore, the high‐throughput phase‐field simulation and machine learning results for shear piezoelectric coefficients (*d*_15_, *d*_31_), stiffness (*c*_11_, *c*_44_), and compliance (*s*_11_, *s*_33_) are presented in Figures S4 and S5 (Supporting Information). It can be seen that the *d*_31_ follows a similar trend to that of *d*_33_.

**Figure 4:**
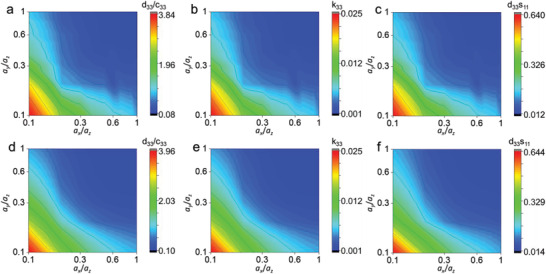
Geometrical effect on the quality factors of nanocomposites, including the phase‐field simulation results of quality factors of a) *d*_33_/*c*_33_, b) electromechanical coupling efficiency *k*_33_, and c) *d*_33_ *s*_11_ with various filler geometries, as well as the machine learning results of d) *d*_33_/*c*_33_, e) *k*_33_, and f) *d*_33_ *s*_11_.

Given that the piezoelectric coefficient and mechanical stiffness both increase upon increasing the length‐to‐width (Figure 3), it is of great significance to seek the specific architecture to simultaneously optimize the piezoelectric response and compliance. Therefore, in order to evaluate the flexibility and high energy conversion efficiency of nanocomposites, a set of quality factors such as *d*_33_/*c*_33_, *k*_33_, and *d*_33_ *s*_11_ are proposed and calculated to characterize the overall performance of the polymers nanocomposites. As shown in **Figure** **4** and Figure S6 (Supporting Information), among the 400 groups of various architectures, the three quality factors all increase upon decreasing the geometric ratios *a*_y_/*a*_z_ and *a*_x_/*a*_z_, indicating that the 1–3 composites with nanopillars parallel to the *z*‐axis possess the optimal effective properties. The expressions of these three quality factors as a function of geometric ratios can also be obtained via machine learning (see Note S1 and Table S2, Supporting Information). Therefore, the architecture of nanopillar is employed for all the following simulation and theoretical analyses. We also investigated the influence of oxide filler size on the piezoelectric properties. As described in Figure S7 (Supporting Information), the effective piezoelectric coefficient (*d*_33_) of sphere fillers (S6) is mainly reversely proportional to the radius (*r*) under a constant volume fraction of 10 vol%. However, the change in piezoelectric coefficient is much less as compared to the case with different filament morphologies.

**Figure 5:**
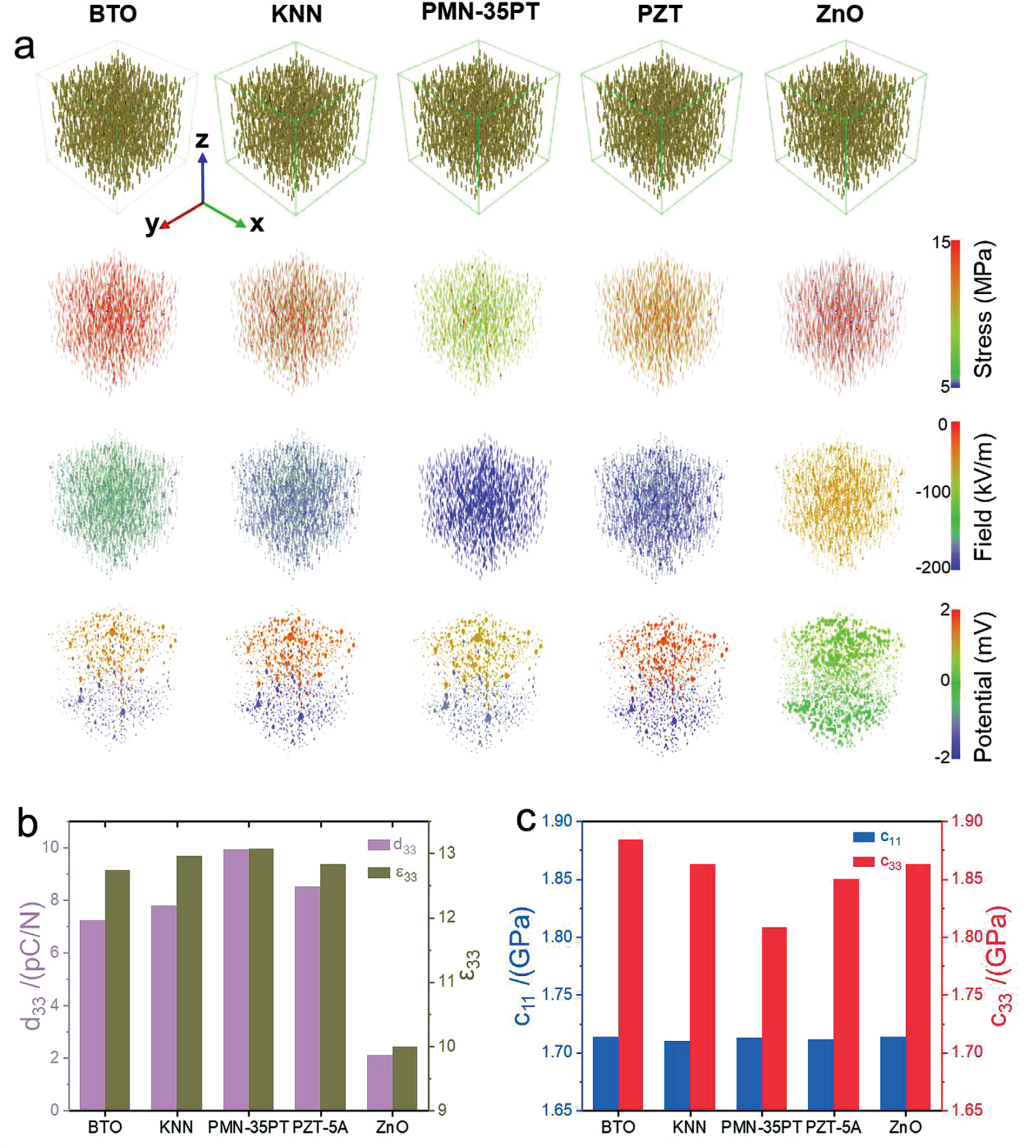
Phase‐field simulation of nanopillar‐ filler‐based nanocomposites with various filler materials. a) Phase‐field simulation results of the stress distribution, piezoelectric field, and electric potential among 5 categories of piezoelectric nanocomposites including BTO/PVA, KNN/PVA, PMN‐35PT/PVA, PZT/PVA and ZnO/PVA. The oxide fillers are at a fixed fraction of 1 vol% with a geometric ratio of (0.1, 0.1). b) Piezoelectric coefficient *d*_33_, relative permittivity *ε*_33_, and c) elastic stiffness *c*_11_ and *c*_33_.

In order to evaluate the impact of filler materials on the output performance of PVA‐polymer nanocomposites, a comprehensive phase‐field simulation is performed to calculate and compare the stress distribution, piezoelectric field, and electric potential among 5 categories of piezoelectric oxides including BTO/PVA, K_x_Na_1‐x_NbO_3_(KNN)/PVA, PMN‐35PT/PVA, PZT/PVA, and ZnO/PVA. The oxide fillers have a fixed geometric ratio of (0.1, 0.1) and a volume fraction of 1 vol% (see **Figure** **5**). The detailed material parameters for different oxide fillers are listed in Table S3 (Supporting Information). It is found that the PMN‐35PT/PVA holds the maximum piezoelectric coefficient and dielectric permittivity when compared with other counterparts. This is due to the inherent nature of the PMN‐35PT oxides with concurrent large electrostrictive coefficient *Q*_33_ and large dielectric permittivity *ε*_33_, which gives rise to a higher *d*_33_ based on the relationship *d*_33_ = 2*P_s_Q*_33_ *ε*_33_.^[^ ^50^ ^]^ Moreover, the PMN‐35PT/PVA nanocomposites hold the smallest mechanical stiffness *c*_33_. In addition, to verify the reliability of the phase‐field simulation, the simulation and experimental results^[^ ^51^, ^52^, ^53^, ^54^ ^]^ are compared in Figure S8 (Supporting Information). For the 0–3 BTO/polymer composites, the piezoelectric response predicted by phase‐field simulation in this study is within the range of the experimentally measured value, which validates the reliability of the current phase‐field model.

**Figure 6:**
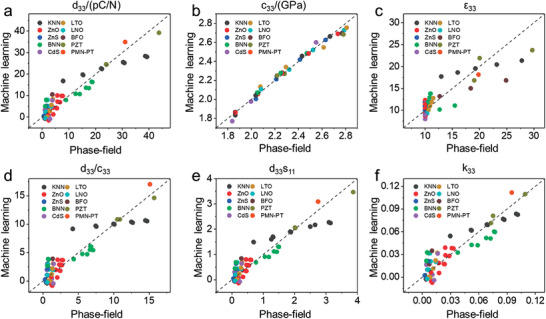
Comparison between material constants from machine learning and those from phase‐field simulation, including a) piezoelectric coefficient *d*_33_, b) stiffness coefficient *c*_33_, c) relative permittivity *ε*_33_, and quality factors d) *d*_33_/*c*_33_, e) *d*_33_ *s*_11_, and f) electromechanical coupling coefficient *k*_33_.

Furthermore, a machine learning strategy is proposed to establish an analytical expression to predict the effective properties of the oxide‐PVA nanocomposites with various filler materials. According to the operational scenario of regression analysis (Figure S9, Supporting Information), three properties of the nanofillers, namely the dielectric permittivity, piezoelectric coefficient, and elastic stiffness are chosen as variables for fingerprints for the machine learning. As illustrated in **Figure** **6**, we train a prediction model to approximate the results of the phase‐field simulation by randomly selecting 70% of the phase‐field simulated results of ten piezoelectric oxide fillers including ((K,Na)NbO_3_ (KNN), PZT, ZnO, (Ba,Na)NbO_3_ (BNN), BiFeO_3_ (BFO), CdS, LiNbO_3_ (LNO), LiTaO_3_ (LTO), ZnS, and PMN‐35PT) as the training dataset. The remaining 30% of phase‐field simulation results are used as the verification dataset to verify the established model. It can be clearly seen from the parity plot that the piezoelectric coefficients of these 10 systems scattered nearby the diagonal dash line with a slope of 1, representing *d*_33_^phase‐field^ = *d*_33_^machine‐learning^, implying that the piezoelectric coefficients for these nanocomposites predicted from the machine learning are in accordance with the results calculated from phase‐field simulations (see Figure 6a), which confirms the validity and reliability of the proposed model. Furthermore, the mechanical stiffnesses with the variety of filler materials are almost aligned along the diagonal dashed line (Figure 6b), showing the consistency and compatibility between the predictive analytical expression and phase‐field simulation. Moreover, the predicted relative permittivity and three quality factors agree well with the corresponding phase‐field simulation results, further validating the applicability and feasibility of the proposed machine learning model (Figure 6c–f). A high coefficient of determination (*R* ^2^) of 0.907 suggests the good performance of our multiple linear regression model. Detailed machine‐learned relationship between the effective properties of the composite and the ceramic filler is given in Method. It can be discovered that the piezoelectric response of the composite shows a positive correlation with the *d*_33_, *c*_11_, *c*_12_, *c*_44_, *d*_15_, and *n* (volume fraction) of the ceramic filament, while it exhibits a negative correlation with *c*_33_ and *ε*_33_ of the ceramic filler. This indicates that the composite material is a complex system where the piezoresponse depends on multiple factors, which can be enhanced by increasing the specific piezoelectric coefficient (*d*_33_), as well as decreasing the dielectric constant (*ε*_33_) and specific elastic stiffness (*c*_33_) of the ceramics.

Finally, to test the generality of the model, we performed the phase‐field simulation of piezoelectric composites based on the PVDF matrix. As shown in Figure S10 (Supporting Information), the 1–3 composite shows better piezoelectric performance as compared to the other composite architectures (i.e., 0–3 composite), consistent with the observations for the PVA‐based composites, showing that the main conclusions of this study can be further extended to the other polymer‐ceramic piezoelectric composites.

## 3 Conclusions

Based on the high‐throughput phase‐field simulation and machine learning, a comprehensive theoretical study is conducted to explore the role of filler materials and composite architecture in determining the piezoelectric and mechanical properties of oxide‐polymer nanocomposites. Among the 400 groups of geometric configurations of oxide fillers examined, nanopillar fillers with the largest length‐to‐width ratio exhibit the maximum piezoelectric coefficient, dielectric permittivity, and mechanical stiffness under given filler volume fractions. Further calculation and regression analysis of quality factors *d*_33_/*c*_33_, *k*_33_, and *d*_33_ *s*_11_ validate that the filler architecture of nanopillar (*a*_x_ */a*_z_ = 0.1, *a*_y_ */a*_z_ = 0.1) simultaneously holds optimal piezoelectric and mechanical attributes for high‐performance flexible composites. Moreover, a machine learning strategy is proposed to establish the analytical expression to predict the effective piezoelectric properties of the PVA‐polymer nanocomposites prepared using various filler materials under diverse volume fractions. This work not only sheds some light on the fundamental mechanism of piezoelectric polymer nanocomposites but also provides an innovative method for optimizing the piezoelectricity of composites for device applications in flexible electronics and energy harvest devices.

## 4 Experimental Section

### Fitting the Surface Formed by the Characteristic Parameters

The Table Curve 3D program is used for mathematical modeling to describe the relationship between the topological shape and orientation of the oxide filler and the characteristic parameters. By comparing the built‐in formulas like *z* = *f* (*x*, *y*), the computer finds the form with the largest correlation coefficient (Figure S7, Supporting Information). The obtained correlation coefficients (*R* ^2^) in the several models are all around 0.95.

### Linear Regression

Certain correlation between the properties of a given material and the properties of the corresponding compound was considered. With the given training data, a predictive multiple linear regression model was trained:

(7) y=α+βx

where **y**  = {*y*_1_,*y*_2_, ⋅⋅⋅, *y*_8_}^T^  refers to the properties (*c*_11_, *c*_12_, *c*_33_, *c*_44_, *ε*_33_, *d*_33_, *d*_31_, *d*_15_) of composite; **x**  = {*x*_1_,*x*_2_, …,  *x*_9_}^T^  includes the corresponding properties of the oxide filler as well as its volume fraction; ***β*** denotes the linear coefficient, and ***α*** is the intercept. The model can be represented in the matrix form as:

(8) $$\left(\begin{array}{c} \hat{c_{11}}\\ \vdots \\ \hat{d_{15}} \end{array}\right)=\left(\begin{array}{ccc} \beta_{11} & \cdots & \beta_{19}\\ \vdots & \ddots & \vdots \\ \beta_{81} & \cdots & \beta_{89} \end{array}\right)\;\left(\begin{array}{c} c_{11}\\ \vdots \\ d_{15}\\ n \end{array}\right)+\left(\begin{array}{c} \alpha_{1}\\ \vdots \\ \alpha_{8} \end{array}\right)$$

where *c*_11_ to *d*_15_ are properties of the oxide filler, *n* is the volume fraction of the ceramic; $\hat{c_{11}}$ to $\hat{d_{15}}$ are properties of the corresponding composite.

Then the square loss was adopted to describe the difference between the predicted value and the true value in the dataset:

(9) $$L\;={\left|\left|Y_{T}-Y_{P}\right|\right|}^{2}\;$$

where *Y*_P_ denotes the characteristic parameter values of composites predicted by the linear regression model and *Y*_T_ refers to the phase‐field simulation values. By minimizing *L*, the ***α*** and ***β*** parameters of the multiple linear regression model were estimated. For example, *d*_33_ are predicted as follows:

(10) $$\begin{array}{ccc} \hat{d_{33}} & = & 0.021c_{11}+0.062c_{12}\;-0.054c_{33}+0.084c_{44}-0.007\varepsilon_{33}\\ & & +\;0.058d_{33}-0.18d_{31}+0.041d_{15}+2.935n\;-\;8.240 \end{array}$$

where the stiffness ***c*** adopts a unit of GPa, the piezoelectric coefficient ***d*** adopts a unit of pC N^−1^, and the dielectric permittivity is unitless. In this expression, the ***d***_**33**_ of the composite can be approximated as a linear combination of several parameters of the ceramic filament. This model summarized by machine learning can be used to estimate the piezoelectric properties of 1–3 polymer/ceramic composites composed of different materials within small amount of ceramic addition. The complete data of the trained model is shown in Table S4 (Supporting Information).

### Statistical Analysis: Pre‐Processing of Data

Since the magnitude difference between the piezoelectric coefficient (C N^−1^) and the stiffness coefficient (Pa) was extremely large (up to 10^22^), the units of the piezoelectric coefficient were adjusted to pC N^−1^ and stiffness coefficient were adjusted to GPa, so that the orders of magnitude of different parameters were similar. It was not only in line with the parameter units commonly used in materials, but also beneficial for machine learning.

### Data Presentation and Sample Size (*n*)

By changing the input parameters of the phase field simulation (the piezoelectric coefficient, the volume fraction of ceramic fillers, etc.), 120 different input–output pairs were obtained (sample size *n *= 120), and 70% of the data (*n*_1_ = 84) were randomly selected for machine learning (training matrix), 30% of the data (*n*_2_ = 36) were used to verify the reliability of the model.

### Statistical Methods Used to Assess Significant Differences with Sufficient Details

The above operations were independently repeated 10 times. The squared loss (*L*) was normalized to the coefficient of determination *R* ^2^, and the average value of *R* ^2^ from 10 trainings was used to evaluate the reliability of the model. The closer the value of *R* ^2^ was to 1, the more accurate the machine learning model was, and it was found that the *R* ^2^ = 0.8563 ± 0.0385.

The coefficient of determination *R* ^2^:

(11) $$R^{2}=\;1-\frac{S_{\mathrm{res}}}{S_{\mathrm{tot}}}$$

(12) $$S_{\mathrm{res}}=\sum_{i}{\left(Y_{T_{i}}-Y_{P_{i}}\right)}^{2}\;=\sum_{i}L_{i}\;$$

(13) $$S_{\mathrm{tot}}=\sum_{i}{\left(Y_{T_{i}}-\bar{Y_{T}}\right)}^{2}\;$$

where *Y*_P_ denotes the characteristic parameter values of composites predicted by the linear regression model and *Y*_T_ refers to the phase‐field simulation values. $\bar{Y_{T}}$ is average of *Y*_T_.

### Software Used for Statistical Analysis

The compilation environment was Python 3.6, and the scikit‐learn module is called for machine learning.

## Conflict of Interest

Co‐author L.‐Q.C. is the owner of Mu‐PRO LLC which licensed the computer codes of generating the phase‐field results from Penn State Research Foundation. All other authors declare no conflict of interest.

## Supporting information

Supporting Information

## Data Availability

The data that support the findings of this study are available from the corresponding author upon reasonable request.
