# Supplementary material for: Optimizing Piezoelectric Nanocomposites by High‐Throughput Phase‐Field Simulation and Machine Learning
Source: Adv Sci (Weinh). 2022 Mar 11;9(13):2105550. doi: 10.1002/advs.202105550 (PMC9069389; doi:10.1002/advs.202105550)
Supplement: Supplementary file 1 — Supporting Information [file ADVS-9-2105550-s001.pdf]

ADVANCED SCIENCE
Open Access

Supporting Information

for *Adv. Sci.*, DOI 10.1002/advs.202105550

Optimizing Piezoelectric Nanocomposites by High-Throughput Phase-Field Simulation and Machine Learning

Weixiong Li, Tiannan Yang, Changshu Liu, Yuhui Huang, Chunxu Chen, Hong Pan, Guangzhong Xie, Huiling Tai, Yadong Jiang, Yongjun Wu, Zhao Kang*, Long-Qing Chen*, Yuanjie Su* and Zijian Hong**

Supplementary Information

Optimizing piezoelectric nanocomposites by high-throughput phase-field simulation and machine learning

Weixiong Li[†], Tiannan Yang[†,], Changshu Liu[†], Yuhui Huang, Chunxu Chen, Hong Pan, Guangzhong Xie, Huiling Tai, Yadong Jiang, Yongjun Wu, Zhao Kang[*], Long-Qing Chen[*], Yuanjie Su[*], Zijian Hong[*]*

Prof. Y. Su, Dr. W. Li, C. Chen, H. Pan, Prof. G. Xie, Prof. H. Tai, Prof. Y. Jiang
School of Optoelectronic Science and Engineering
University of Electronic Science and Technology of China
Chengdu 610054, P.R. China
E-mail: yjsu@uestc.edu.cn (Y.S.)

Dr. T. Yang, Prof. L.Q. Qing
School of Materials Science and Engineering
The Pennsylvania State University
University Park 16802, USA
E-mail: tuy123@psu.edu (T.Y.), lqc3@psu.edu (L.C.)

Prof. Z. Kang, Dr. C. Liu
School of Computer Science and Engineering
University of Electronic Science and Technology of China
Chengdu 610054, P.R. China
E-mail: zkang@uestc.edu.cn (Z.K.)

Dr. Y. Huang, Prof. Y. Wu, Prof. Z. Hong
Lab of Dielectric Materials
School of Materials Science and Engineering
Zhejiang University
Hangzhou 310027, P.R. China
E-mail: hongzijian100@zju.edu.cn (Z. H.)

Keywords: piezoelectric, nanocomposite, high-throughput phase-field simulation, machine learning

Table S1. Materials constants of BTO and PVA.

BTO:

relative dielectric permittivity $\boldsymbol{\varepsilon}_r$

$$\begin{bmatrix} 1200 & & \\ & 1200 & \\ & & 1200 \end{bmatrix}$$

piezoelectric coefficient **d**

$$\begin{bmatrix} 0 & 0 & 0 & 0 & 270 & 0 \\ 0 & 0 & 0 & 270 & 0 & 0 \\ -79 & -79 & 191 & 0 & 0 & 0 \end{bmatrix} \text{(pC/N)}$$

elastic stiffness c

$$\begin{bmatrix} 178 & 96.4 & 96.4 & 0 & 0 & 0 \\ 96.4 & 178 & 96.4 & 0 & 0 & 0 \\ 96.4 & 96.4 & 178 & 0 & 0 & 0 \\ 0 & 0 & 0 & 122 & 0 & 0 \\ 0 & 0 & 0 & 0 & 122 & 0 \\ 0 & 0 & 0 & 0 & 0 & 122 \end{bmatrix} \text{(GPa)}$$

PVA:

relative dielectric permittivity $\boldsymbol{\varepsilon}_r$

$$\begin{bmatrix} 10 & & \\ & 10 & \\ & & 10 \end{bmatrix}$$

piezoelectric coefficient **d**

$$\begin{bmatrix} 0 & 0 & 0 & 0 & 0 & 0 \\ 0 & 0 & 0 & 0 & 0 & 0 \\ 0 & 0 & 0 & 0 & 0 & 0 \end{bmatrix} \text{(pC/N)}$$

elastic stiffness **c**

$$\begin{bmatrix} 1.69 & 0.87 & 0.87 & 0 & 0 & 0 \\ 0.87 & 1.69 & 0.87 & 0 & 0 & 0 \\ 0.87 & 0.87 & 1.69 & 0 & 0 & 0 \\ 0 & 0 & 0 & 0.41 & 0 & 0 \\ 0 & 0 & 0 & 0 & 0.41 & 0 \\ 0 & 0 & 0 & 0 & 0 & 0.41 \end{bmatrix} \text{(GPa)}$$

Note S1. Expressions of quality factors

In the fitting, the results are dissimilar by using different ranges of data. This is because in the entire 20×20 range, the magnitude of the data is very different. The fitting will choose the case where the average error is small, but this will cause the error of (0.1, 0.1) and nearby points (their values to change drastically) become larger. So here, we choose the geometric ratio from 0.1 to 1, and do another fitting with 10×10 points. For the fitting function in a small area, the error is reduced, but the applicable range is also reduced.

The range of 10×10 in **Fig. 4**:

$$d_{33}/c_{33} = \frac{a+b\ln x+c\ln^2 x+d\ln y+e\ln^2 y+f\ln^3 y}{1+g\ln x+h\ln^2 x+i\ln y+j\ln^2 y} \quad (1)$$

$$k_{33} = \frac{a+b\ln x+c\ln^2 x+d\ln^3 x+e\ln y+f\ln^2 y}{1+g\ln x+h\ln^2 x+i\ln y+j\ln^2 y} \quad (2)$$

$$d_{33}s_{11} = \frac{a+b\ln x+c\ln^2 x+d\ln^3 x+e\ln y+f\ln^2 y}{1+g\ln x+h\ln^2 x+i\ln y+j\ln^2 y} \quad (3)$$

The range of 20×20 in **Fig. S6**:

$$z = \frac{a+c\ln x+e\ln y+g\ln^2 x+i\ln^2 y+k\ln x\ln y}{1+b\ln x+d\ln y+f\ln^2 x+h\ln^2 y+j\ln x\ln y} \quad (4)$$

This expression is exactly the same for the three quality factors, so z represents d_{33}/c_{33}, k_{33}, and $d_{33}s_{11}$ respectively.

The specific value of the coefficients in the expressions are in **Table S2**.

Table S2. Coefficients for expressions of the quality factor

In the range of 10×10:

	**a**	**b**	**c**	**d**	**e**
d_{33}/c_{33}	0.112824	0.095863	0.080419	-0.037756	-0.093693
k_{33}	0.000683	0.000597	0.000487	-0.000494	-0.000241
$d_{33}s_{11}$	0.015309	-0.007259	-0.015120	-0.015243	0.005455
	**f**	**g**	**h**	**i**	**j**
d_{33}/c_{33}	-0.080056	0.454427	0.085712	0.285105	0.089104
k_{33}	-0.000589	0.468187	0.089616	0.279146	0.086415
$d_{33}s_{11}$	0.004178	0.119441	0.058024	0.529040	0.078339

In the range of 20×20:

	**a**	**b**	**c**	**d**	**e**	**f**
d_{33}/c_{33}	0.101357	0.394589	-0.001939	0.402369	-0.001294	0.004402
k_{33}	0.000611	0.397090	-0.000010	0.403412	-0.000007	0.005104
$d_{33}s_{11}$	0.015706	0.373282	-0.000543	0.422551	-0.000097	-0.003383
	**g**	**h**	**i**	**j**	**k**	
d_{33}/c_{33}	-0.001493	0.008120	-0.001224	0.150060	0.004721	
k_{33}	-0.000009	0.008285	-0.000007	0.150417	0.000028	
$d_{33}s_{11}$	-0.000323	0.011846	-0.000518	0.153484	0.000993	

Table S3. Material parameters for different ceramic fillers

	c_{11} (GPa)	c_{12} (GPa)	c_{33} (GPa)	c_{44} (GPa)	ε_{33}	d_{33} (pC/N)	d_{31} (pC/N)	d_{15} (pC/N)
**ZnO**	209.7	121.0	211.2	42.4	10.2	11.67	-5.43	-11.34
**KNN**	136.2	86.2	98.5	22.8	2000	380.0	-140	690.00
**PZT**	121.0	77.0	111.0	21.0	1700	374.0	-171	584.00
**PMN-35 PT**	115.0	103.0	103.0	69.0	8200	2820	-1300	146.00
**BTO**	178.0	96.4	178.0	122.0	1200	191.0	-79.00	270.00
**ZnS**	120.4	69.2	127.6	22.8	8.7	3.23	-1.13	-2.80
**CdS**	90.7	58.1	93.8	15.0	9.5	10.3	-5.00	-14.00
**PZT**	126.0	67.9	117.0	22.2	3400	593.0	-274.00	741.00
**LNO**	203.0	53.0	245.0	60.0	29.0	6.0	-1.00	68.00
**LTO**	233.0	53.0	275.0	94.0	43.0	8.0	-2.00	26.00
**BFO**	239.0	144.0	164.0	41.0	1000	18.0	-12.67	27.60
**BNN**	239.0	104.0	135.0	65.0	32.0	37.0	-7.00	42.00

Table S4. Complete data of the trained model

β =

-3.18E-4	2.12E-4	1.52E-4	1.74E-3	-2.70E-6	-2.40E-5	-6.92E-5	5.06E-6	2.57E-2
-1.44E-3	8.64E-4	3.26E-4	1.38E-3	-2.03E-6	-9.56E-6	-3.57E-5	2.36E-6	1.18E-2
6.04E-2	-3.13E-2	2.93E-2	1.40E-2	3.06E-6	-3.18E-4	-5.48E-4	1.14E-4	2.07E-1
1.09E-3	-7.47E-4	2.12E-4	6.87E-4	2.40E-7	3.05E-6	6.85E-6	5.00E-7	1.00E-2
-6.63E-1	2.14E+0	-9.15E-1	4.31E+0	3.79E-3	-4.33E-4	1.97E-2	6.25E-3	9.09E-1
2.10E+0	6.22E+0	-5.38E+0	8.41E+0	-6.69E-3	5.85E-2	-1.79E-1	4.11E-2	2.93E+0
1.16E-2	-2.20E+0	1.89E+0	-3.49E+0	1.80E-3	2.20E-2	6.27E-2	-1.26E-2	-1.11E+0
1.55E+0	-2.45E+0	-5.08E-2	-4.50E-1	5.36E-4	1.35E-3	4.98E-3	7.86E-4	1.21E-1

α =

1.686
0.869
1.499
0.406
6.834
-8.239
1.870
-0.869

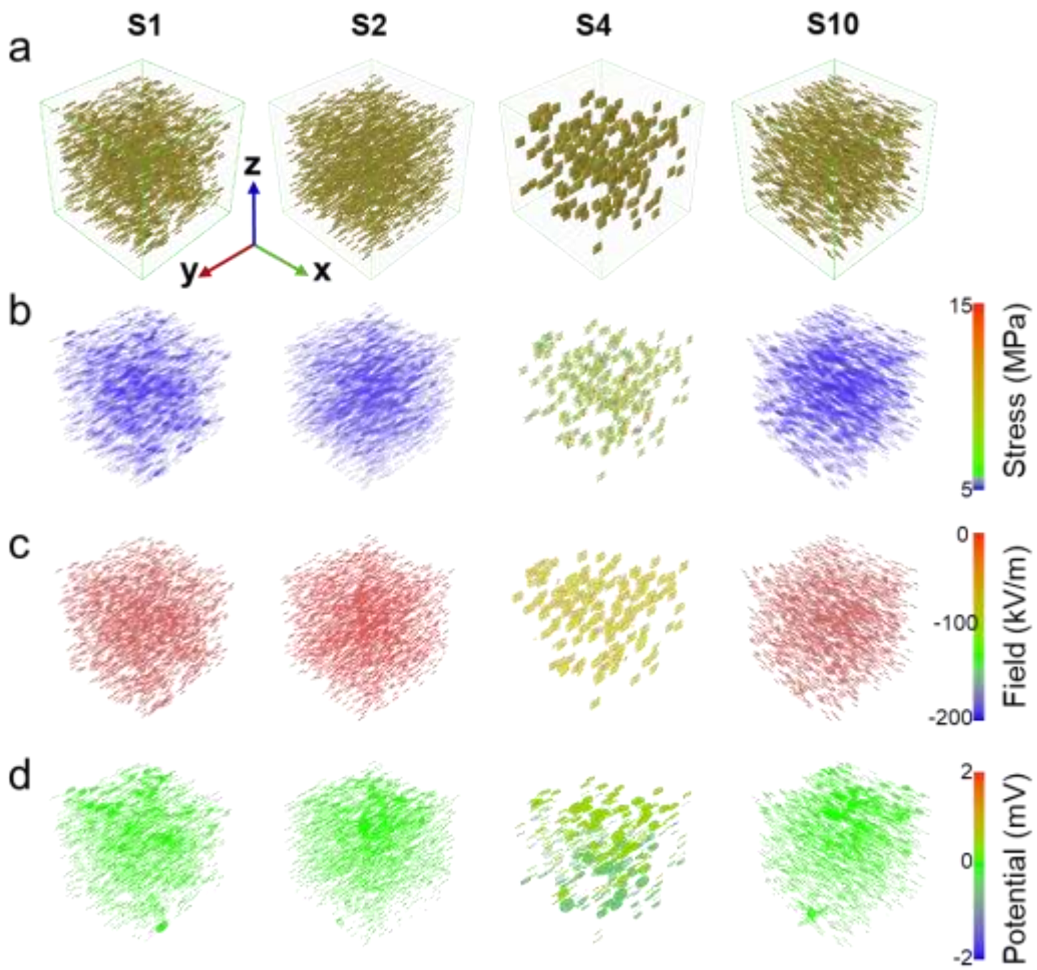

Figure S1. Phase-field simulation results of (a) four types of nanocomposites with randomly aligned nanofillers with diverse shape and orientation. Their corresponding (b) stress distribution, (c) electric field and (d) electric potential in response to the applied stress of 1 MPa along the z-axis.

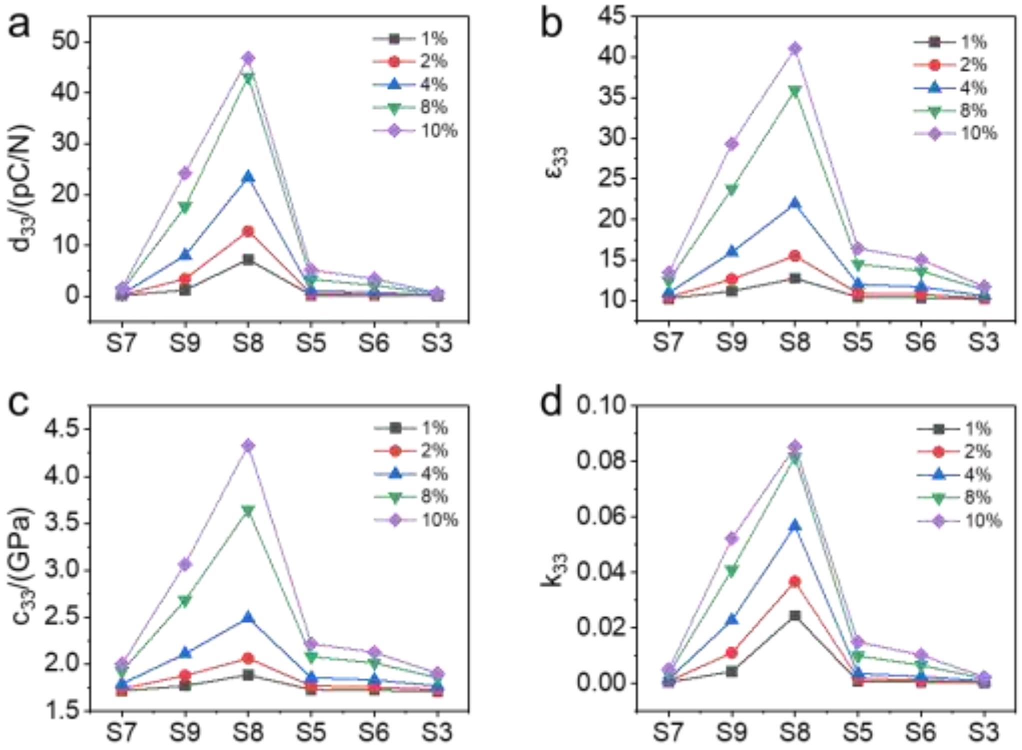

Figure S2. The dependence of material constants d_{33}, ε_{33}, k_{33}, and c_{33} on the type of geometric architectures (S7, S9, S8, S5, S6, S3) under given volume fractions.

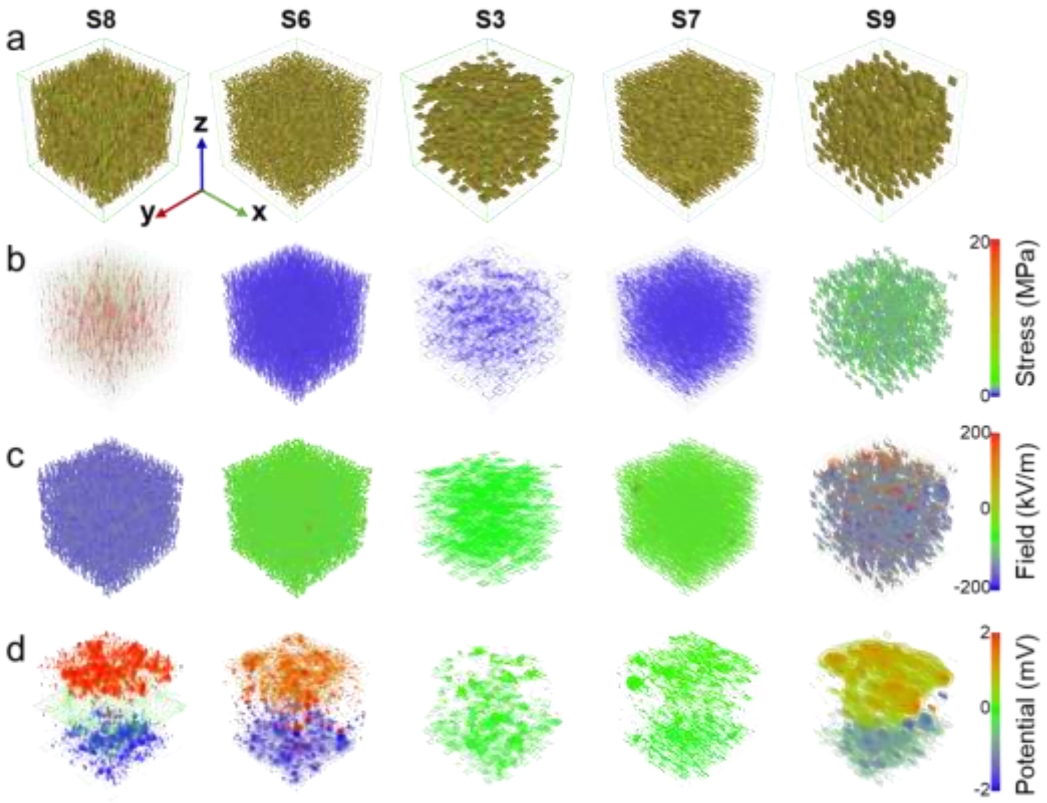

Figure S3. Phase-field simulation of 5 types of nanocomposites with different filler configurations at a 4 vol% filler fraction. (a) Structure of the selected types of nanocomposites. (b-d) Their corresponding (b) stress, (c) electric field, and (d) electric potential distribution in response to an applied stress of 1 MPa along the z-axis.

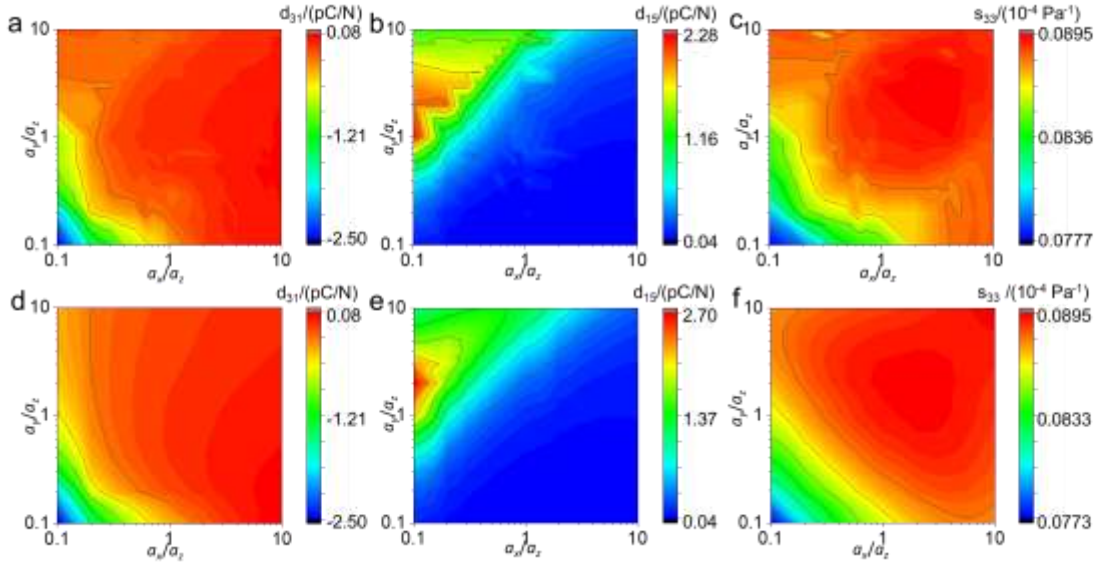

Figure S4. The high-throughput simulation results of the whole dataset (20×20) of the nanocomposites, (a) piezoelectric coefficient (d_{31}), (b) piezoelectric coefficient (d_{15}) and (c) compliance coefficient (s_{33}). The machine learning results of (d) piezoelectric coefficient (d_{31}), (e) piezoelectric coefficient (d_{15}), and (f) compliance coefficient (s_{33}).

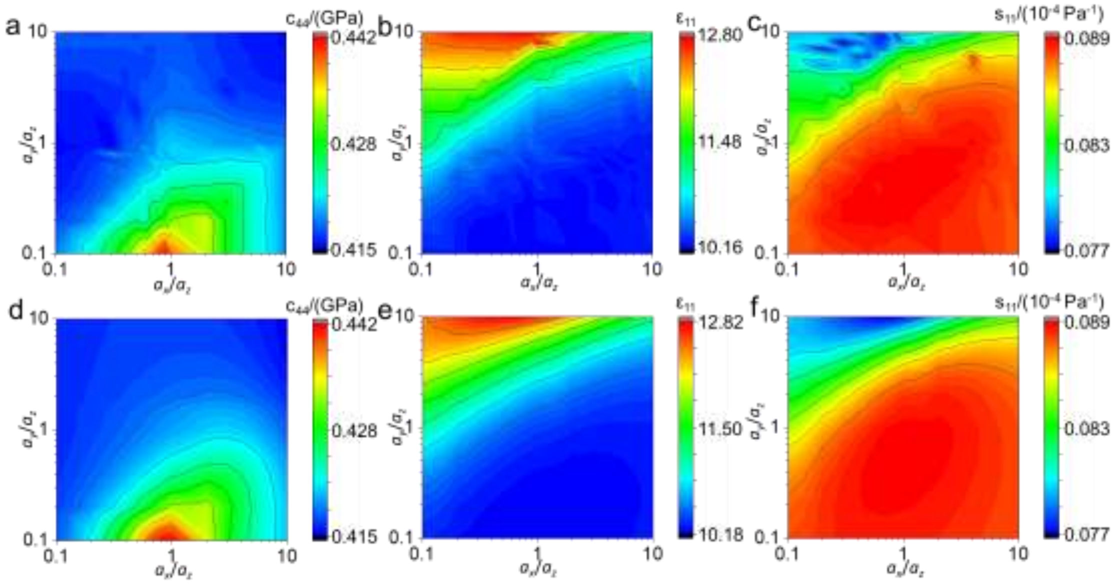

Figure S5. The high-throughput simulation results of the whole dataset (20×20) of the nanocomposites, (a) elastic stiffness (c_{44}), (b) relative permittivity (ε_{11}) and (c) compliance coefficient (s_{11}). The machine learning results of (d) elastic stiffness (c_{44}), (e) relative permittivity (ε_{11}) and (f) compliance coefficient (s_{11}).

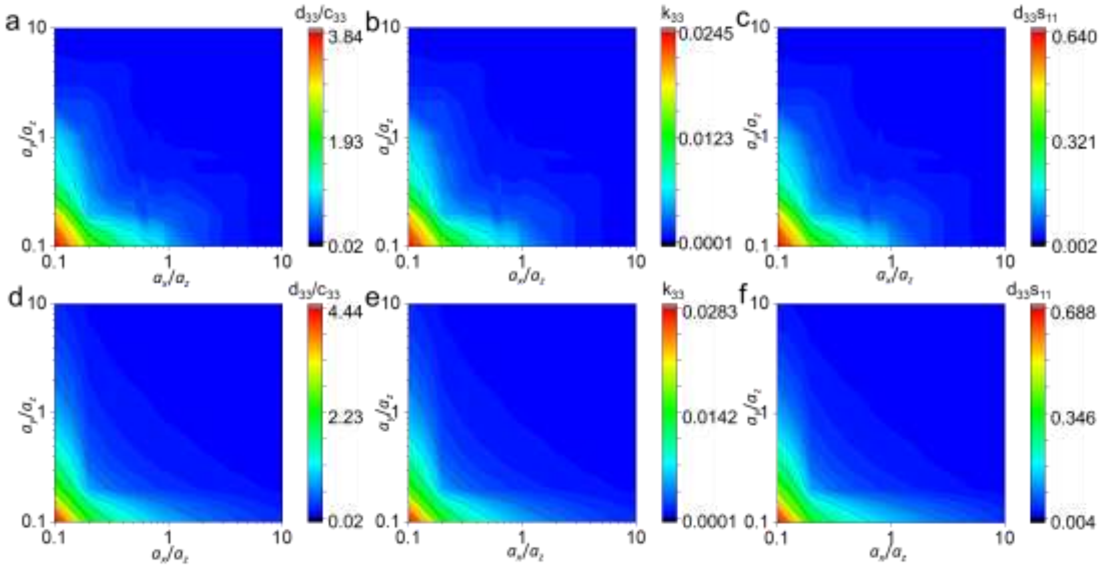

Figure S6. A set of quality factors including (a) d_{33}/c_{33}, (b) electromechanical coupling efficiency (k_{33}), and (c) $d_{33}s_{11}$ of the whole dataset (20×20) of the nanocomposites. The machine learning results of (d) d_{33}/c_{33}, (e) k_{33}, and (f) $d_{33}s_{11}$ along with the geometry variation.

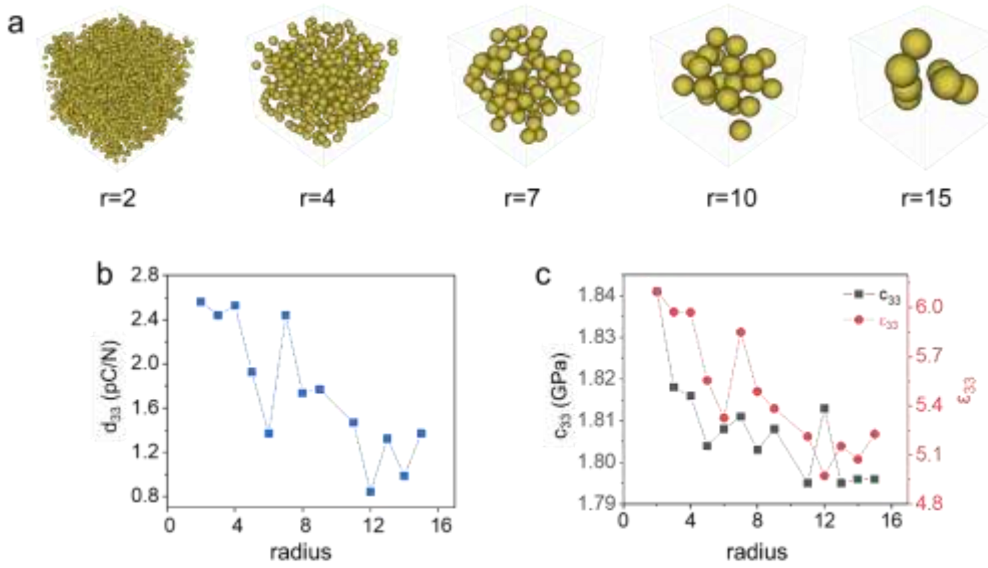

Figure S7. PMN-35PT/PVA phase field simulation results. The absolute size of ceramic particles increases, and the parameters obtained by phase field simulation. (a) A schematic diagram of the morphology of the composite structure as the radius increases. (b) Piezoelectric coefficient d_{33}, (c) stiffness coefficient c_{33} and relative permittivity ε_{33}.

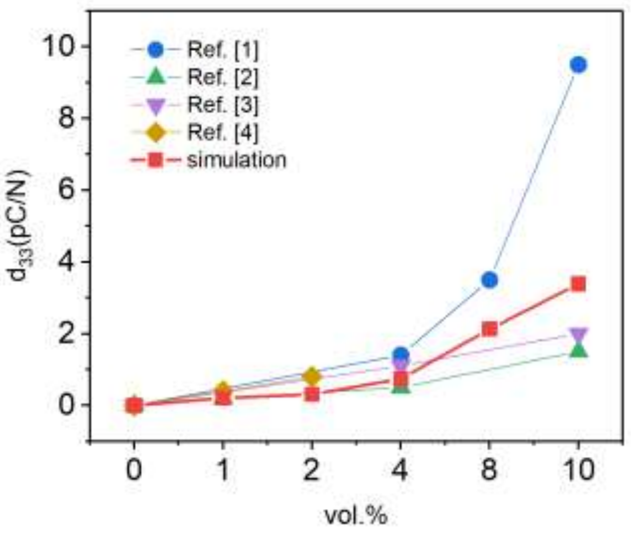

Figure S8. Comparison of the d_{33} of the BTO-polymer experimental results [R1-R4] with phase-field simulations.

[R1] Cho, Y. et al., $BaTiO_3$@ PVDF-TrFE nanocomposites with efficient orientation prepared via phase separation nano-coating method for piezoelectric performance improvement and application to 3D-PENG. *Chem. Eng. J.* **2022**, 427, 131030.

[R2] Capsal, J. F. et al., Nanotexture influence of $BaTiO_3$ particles on piezoelectric behaviour of PA 11/$BaTiO_3$ nanocomposites. *J. Non-Cryst. Solids* **2010**, 356, 629.

[R3] Hua, Z. et al., Preparation, structure, and property of highly filled polyamide 11/$BaTiO_3$ piezoelectric composites prepared through solid-state mechanochemical method. *Polym. Compos.* **2019**, 40, E177.

[R4] Guo, J. et al., PA1111/$BaTiO_3$ nanocomposites with surprisingly enhanced piezoelectricity at low filler content via in-situ compositing process. *Compos. Sci. Technol.* **2021**, 209, 108796.

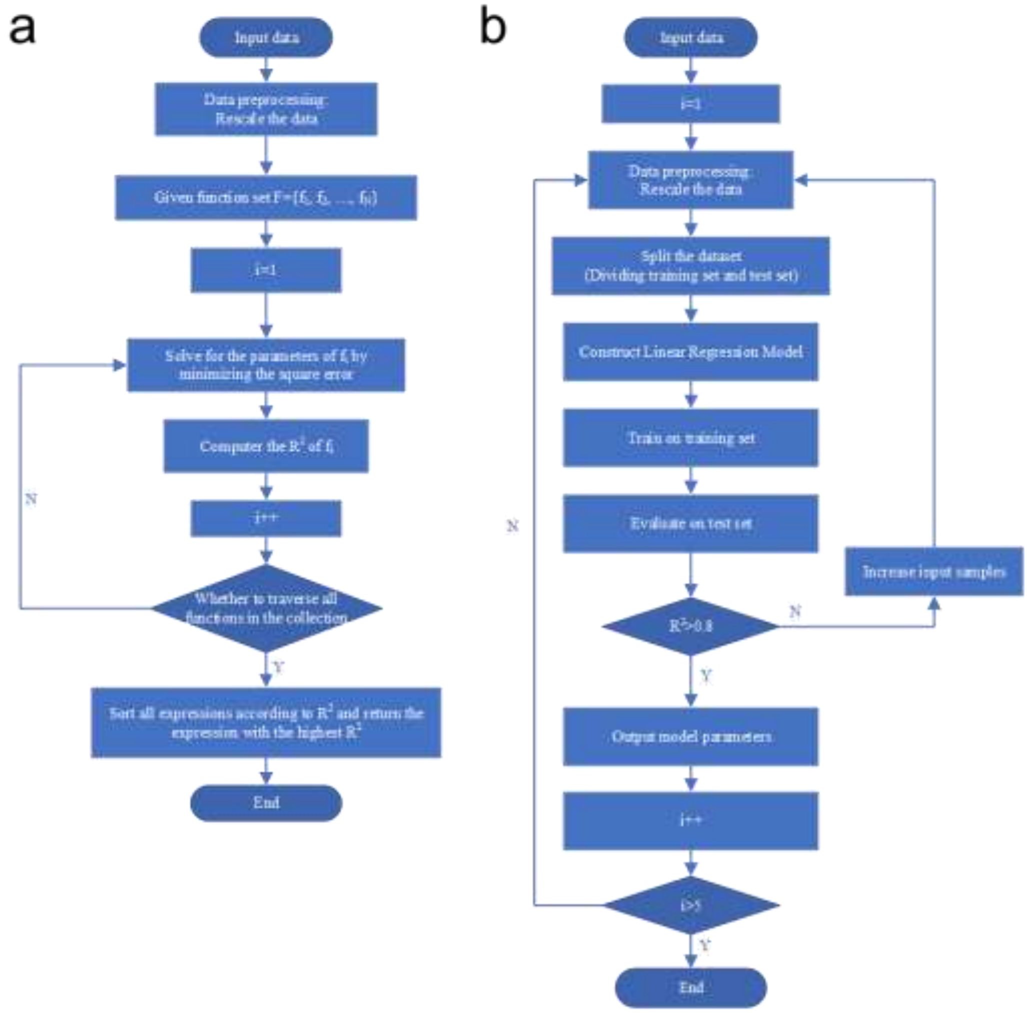

Figure S9. Flow chart of (a) fitting the surface formed and (b) linear regression.

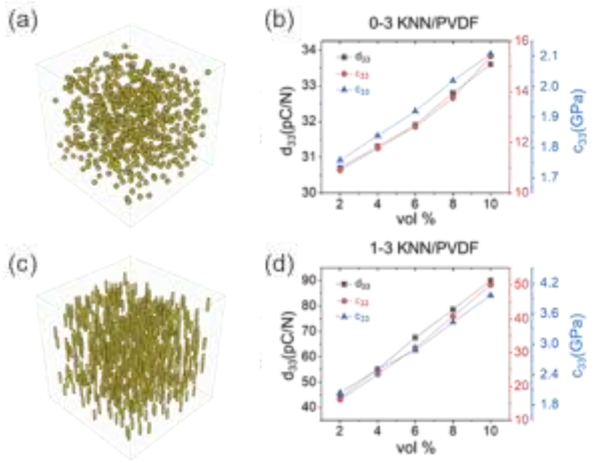

Figure S10. Phase-field simulations of the KNN/PVDF composites. (a) S6 structure, (b) piezoelectric coefficient (d_{33}), relative permittivity (ε_{33}) and stiffness coefficient (c_{33}) of the 0-3 KNN/PVDF composite. (c) S8 structure, (d) d_{33}, ε_{33} and c_{33} of the 1-3 KNN /PVDF composite. It can be discovered that the 1-3 composite show much higher piezoresponse as compared to the 0-3 composite.
